# Reduction of Cd Uptake in Rice (*Oryza sativa*) Grain Using Different Field Management Practices in Alkaline Soils

**DOI:** 10.3390/foods12020314

**Published:** 2023-01-09

**Authors:** Obey Kudakwashe Zveushe, Qin Ling, Xing Li, Sumbal Sajid, Víctor Resco de Dios, Farhan Nabi, Ying Han, Faqin Dong, Fang Zeng, Lei Zhou, Songrong Shen, Wei Zhang, Zhi Li

**Affiliations:** 1 School of Life Science and Engineering, Southwest University of Science and Technology, Mianyang 621010, China; 2 School of Environment and Resource, Southwest University of Science and Technology, Mianyang 621010, China; 3 Key Laboratory of Solid Waste Treatment and Resource Recycle, Southwest University of Science and Technology, Mianyang 621010, China; 4 Department of Crop and Forest Sciences, University of Lleida, 25003 Lleida, Spain; 5 Joint Research Unit CTFC-AGROTECNIO, Universitat de Lleida, 25003 Lleida, Spain; 6 Fundamental Science on Nuclear Wastes and Environmental Safety Laboratory, Southwest University of Science and Technology, Mianyang 621010, China; 7 Center of Analysis and Testing, Southwest University of Science and Technology, Mianyang 621010, China; 8 Chengdu Defei Environmental Engineering Co., Ltd., Chengdu 610041, China

**Keywords:** rice, silicon fertilizer, cadmium bioavailability, soil amendment, cadmium toxicity

## Abstract

Cadmium contamination and toxicity on plants and human health is a major problem in China. Safe rice production in Cd-contaminated alkaline soils, with acceptably low Cd levels and high yields, remains an important research challenge. To achieve this, a small-scale field experiment with seven different soil amendment materials was conducted to test their effects performance. Two best-performing materials were selected for the large-scale field experiment. Combinations of humic acid, foliar, and/or soil silicon fertilization and deep or shallow plowing were designed. It was found that the combination, including humic acid, soil and foliar silicate fertilization, and shallow plowing (5–10 cm), produced the most desirable results (the lowest soil bioavailable Cd, the lowest grain Cd concentrations, and the highest grain yield). Rice farmers are therefore recommended to implement this combination to attain high grain yield with low Cd concentrations in alkaline soils.

## 1. Introduction

Cadmium (Cd) is a major environmental pollutant and one of the most toxic metals in the environment. Environmental contamination with Cd primarily stems from the parent rock [1] and also through anthropogenic activities, including agriculture, mining, metallurgy, and manufacturing [2,3]. Cd is a non-essential element that has high rates of soil-to-plant transference relative to other non-essential elements [4,5], and certain plant species accumulate large amounts of Cd from low Cd-content soils [6]. In the past five decades, global Cd emissions have reached a total of 2.2 × 10^5^ tons, and Cd pollution is becoming increasingly severe [7,8]. Cd enters the plant via the roots through the pathways of other divalent cations such as calcium (Ca) [9]. When inside the plant system, Cd toxicity affects physiological and biochemical processes, such as seed germination, photosynthesis, nutrient uptake, or growth and yield [10,11,12,13]. Research has shown that Cd can lead to high economic losses in the production of food crops such as rice (*Oryza sativa* L.) and maize (*Zea mays*) [14].

As mentioned before, sources of Cd contamination are both natural and anthropogenic. Cd can be introduced to the soil through the use of Cd-contaminated irrigation water [15]. When Cd accumulates in either soil or water, it exists in two different forms (bioavailable form or non-bioavailable form). On the one hand, aside from being transported into groundwater through permeable soil layers, the bioavailable Cd can be absorbed by plants through root uptake, thus entering the food chain and leading to various plants, animals, and human health problems [16,17]. Some studies have reported Cd in plant tissues and the concentrations of their bioavailable forms in respective soils [18,19]. Non-bioavailable Cd, on the other hand, is the form in which the Cd is not readily available for absorption. As a result, non-bioavailable Cd accumulation in the soil does lead to high levels soil Cd concentration levels but has less toxicity to plants since non-available Cd cannot be absorbed by the plant. It is worth mentioning that the bioavailability of Cd in the soil can potentially be mobilized by precipitation, adsorption, or complexation with organic amendments [20]. Increasing the rhizosphere pH has also been proven to be an effective way of stabilizing bioavailable Cd in the soil [21]. Quantification of the Cd in bioavailable forms can provide useful information about the metal concentrations in plant tissues, either bioaccumulated in roots or translocated to the above-ground parts of economic importance, such as grain in crops like rice.

Rice is one of the most important crops globally. In Asia, more than 50% of the population consumes rice on a daily basis. In addition to being an important food source, rice can also be used as raw material for developing a variety of products, including wine or sugar [22,23,24]. Under the increasing pressure to feed an ever-growing population, high-yield cultivars such as hybrid rice have been developed. Currently, 45% of China’s 0.70 million m^2^ rice cultivation farmland is occupied with hybrid rice, but it can reach 95% of the total land in some Provinces like Sichuan (in Southwest China). The annual yield of rice in China is about 700 million tons, and about 12 million tons are polluted by heavy metals, including around 1.5 million tons of rice polluted by cadmium (Cd) [25,26].

One of the major challenges in rice production and consumption to the Chinese population is heavy metal (Cd in particular) contamination of both the farmland and the irrigation water [27,28,29]. On a countrywide scale, over 10% of the rice grains collected from China’s rice markets exceeded the national food standards for rice grain Cd [21,30]. The first case of rice contamination with Cd was in 1982, and the contaminated rice was later referred to as “Cd rice” from paddy fields in Taoyuan county, China (Taiwan), and subsequent discoveries of rice contaminated with Cd were reported in many areas in Taiwan [14]. The irrigation water used in these rice paddies was contaminated by wastewater containing high Cd concentrations from nearby chemical factories producing polyvinyl chloride stabilizers. Compared with other crops, rice has a greater ability to uptake Cd and water from the soil. Paddy plants are grown under flooded conditions, which enable toxic elements to be assimilated by plants [31]. The same problem of high Cd concentrations in rice grains is still prevalent in China [32,33].

The current study is motivated by a governmental study documenting that the concentrations of Cd in paddy soils and rice grains in some areas (including the experimental areas in this research) from Sichuan were 0.3–2.8 mg kg^−1^ and 0.26–0.39 mg kg^−1^ respectively, which is substantially beyond the national standard for safe values (GB 15618-2018 and GB 2762-2017) [34]. To solve this problem, it was proposed that there is a need to examine the best way to produce safe rice without yield penalties. Regarding the application of Cd remediation strategies that ensure both high grain yields as well as a safe food supply, there are different alternatives such as soil amendments [35,36,37], selecting rice cultivars with low Cd accumulation rates [38,39], changes in fertilization [40,41,42,43], altering the soil redox through irrigation [44,45,46] or dilution of soil Cd in the plow layer by practicing deep plowing [47,48].

Special soil amendments are usually designed to alter soil pH and transform exchangeable heavy metals into organically bound and residual forms through a series of processes such as adsorption, precipitation, complexation, ion exchange, and redox, thus reducing their bioavailability and solubility [35,49,50]. Different types of soil amendment materials used to reduce the bioavailability and uptake of Cd include the use of biochar, humic acid, sepiolite, silicate fertilizers, phosphate fertilizers, and sulfate fertilizers [51,52]. For instance, among the passivator materials, biochar and sulfate fertilizers are commonly used amendments for the reduction of Cd in agricultural soils to ensure the safe production of rice [53,54,55]. Compared to inorganic fertilizers, biochar-based fertilizers are eco-friendly and are cheap to buy [53]. Adding biochar to the soil can also enhance the soil’s acidity by increasing soil pH and physical structure, enhancing the water-holding capability and soil fertility, thus ultimately enhancing final crop yield [56]. Furthermore, amending the soil with humic acid has been reported to reduce the concentrations of soluble heavy metals and organic forms of many heavy metals, including Cd. The use of humic acid in rice fields has also been reported to improve rice growth and grain yield [57]. Moreover, the use of sepiolite (a clay mineral) as a soil amendment material has been reported to be effective in reducing Cd mobility and bioavailability in the soil, hampering Cd transfer in the soil-plant system and then ameliorating the risk to underground waters and/or receptor organisms [52]. Furthermore, sepiolite has been reported to improve soil fertility, enhancing rice growth and yield [51,52].

In the current study area, the soils are alkaline, with a pH relatively high (average of 7.8) and low organic matter content (data supplied by the local agricultural department). The amendments currently available on the market (i.e., lime, calcium magnesium phosphate fertilizer) have been widely used in acidic soils, where they can greatly reduce soil bioavailable Cd concentrations [42,46,58]. However, the effects of these amendments on alkaline soils, such as the ones from southwestern Sichuan, are poorly understood. Changes in the fertilization regime and application methods (foliar or basal application) are also a strategy to consider. Rice is a silicon-loving plant [59]. Studies have shown that foliar application of silicon (Si) based fertilizers significantly increased the dry weight of grains and shoots in rice grown in Cd-contaminated soil. The total accumulation of Cd in rice grains also decreased with the application of Si [40]. The foliar spraying and soil addition of silicon fertilizer can facilitate the deposition of Cd^2+^ in the cell wall of roots, stems, and leaves to form a Si-Cd complex, consequently reducing the migration of Cd to rice grains [60,61,62]. Furthermore, sulfate fertilizers have also been reported to reduce bioavailable Cd percentage and increase the stable Cd percentages in the soil. Cd transfer from rice roots to shoots was also found to be reduced by the application of sulfate fertilizers in Cd-contaminated soils [63].

Another strategy is to use deep plowing [64]. Indeed, if the Cd concentration in the bottom soil is low and the Cd concentration in the tillage layer is high, Cd concentrations could, at least potentially, be shifted from shallow to deep soil layers, and Cd in the tillage layer could be diluted through soil mixing when deep plowing. In some parts of Southwest China, there is a problem of high Cd contamination in farmlands, leading to high Cd concentrations in rice grains and current farming practices show low efficiency in soil remediation. Adopting cost-effective and environmentally sustainable methods for high-yield production of low Cd rice grains is an important goal for rice farmers in Cd-contaminated soils.

To achieve this, an experiment was carried out on Cd-contaminated farmlands of 5 towns in Jinyang county, Southwest Sichuan, China, to establish an efficient method to produce high rice grain yield that meets safety standards. More specifically: (1) a study was carried out to elucidate the effects of 7 different soil amendments on the remediation of Cd-contaminated alkaline paddy soils, Cd accumulation in rice grain, and rice grain yield; (2) the two best-performing amendment treatments together with plowing depths method (shallow, and deep plowing) and fertilization application methods (soil and foliar application) in a large-scale field experiment were then implemented in a large-scale field study to test their potential under a bigger and more realistic setting.

It was hypothesized that (1) combining the different soil amendments would lead to more Cd stabilization relative to the use of one type of material; (2) combining foliar and soil-applied fertilizers reduce Cd uptake by rice as the foliar-applied nutrients will reduce the affinity of rice to uptake nutrients via the root system which also absorbs Cd from the soil, and (3) deep plowing reduced Cd toxicity on rice plants and also Cd uptake by rice plants as the soil Cd will be diluted by fresh soil when you mix the topsoil and the soil from deep under the surface which is less contaminated with Cd.

## 2. Materials and Methods

### 2.1. Study Area

This research was carried out in Jinyang county, which occupies 841 km^2^ in Southwest Sichuan province, China. The area climate is warm and humid with annual average temperature and rainfall of 17 °C and 1026 mm, respectively (Provided by Local Meteorological Bureau).

### 2.2. Soil Characterization of Physicochemical Properties

To characterize the initial and final soil physiochemical properties, a randomly placed soil core (5 cm × 20 cm) was collected within each of the 16 plots in April (application of soil amendments) and September (at experiment termination). Five sites in each plot were selected to collect soil samples and pooled together as one sample, three replicates for each treatment, and the soils were passed through a 2 mm-mesh size sieve to remove roots and debris, air-dried under a shade, and stored in dry paper bags at room temperature for further analyses. Soil pH was determined in a 1:2.5 (soil: distilled water) solution following the method by Soil pH was determined following the method described by Anderson and Ingram [65], with only minor modifications. The modified procedure includes using 10 ± 0.1 g of soil instead of 20 ± 0.1 g of soil and adding 25 mL of distilled water instead of 50 mL. Air-dried soil samples were prepared and analyzed in triplicates. All water-soil mixtures were agitated for 10 min on a rotor shaker (SHA-B Laboratory Thermostatic Constant-Temperature Shaker, Changzhou, China) at 250 rpm. Then the mixtures were left to stand for 30 min, then stirred again for 2 min before measuring the pH. The pH of the supernatant was measured and noted when the reading stabilized (0.1 unit per 30 s or 0.02 units per 5 s) Van Reeuwijk [66] using a pH meter (Mettler Toledo 30266628 Fiveeasy Model FP20 Benchtop pH Meter, Columbus, OH, USA).

Soil exchangeable NH_4_^+^ and available NO_3_^−^ were determined using the method described by Keeney and Nelson [67]. Briefly, 5 g of soil was mixed with an extraction solution (1M potassium chloride) at a ratio of 1:10 (m/v) in an extraction bottle. The bottle was tightly sealed and then put on a shaker for 60 min, then filtered, and the filtrate was collected for the determination of nitrogen fractions. Measurements of NH_4_^+^ and NO_3_^−^ were done using continuous flow analysis against known standard solutions. Soil-available phosphorus P and exchangeable cations (K^+^, Ca^2+^, Mg^2+^, Na^+^, Fe^2+^, and Mn^2+^) were extracted using Mehlich’s No. 1 double acid solution [68]. In short, a mixture of 0.05M hydrochloric acid (HCl) + 0.025M sulphuric acid (H_2_SO_4_) was used to dissolve adsorbed P, K^+^, Ca^2+^, Mg^2+^, Al^3+^, Na^+^, Fe^2+^, and Mn^2+^. 5 g of dry soil were mixed with the extraction solution at a ratio of 1:10 (m/v). The samples were shaken and centrifuged at 10000 rpm for 10 min; then, the supernatants were filtered through a 0.22-µm filter paper. Soil-available P in the soil was then determined using Ultraviolet-visible spectrophotometry (Lambda 25, Perkin Elmer, Shelton, CT, USA) after a blue color development [68], whereas exchangeable cations were determined using an Atomic Absorbance Spectrophotometer (AAnalyst 800, Perkin Elmer, Shelton, CT, USA). Soil exchangeable Al^3+^ was determined using the acid-base titration method described by Rowen [69]. The EC was determined by summing the charge equivalent of exchangeable K^+^, Ca^2+^, Mg^2+^, Na^+^, Mn^2+^, Fe^2+^, Al^3+^, and H^+^ ions for soil samples with a pH lower than 6.5 and soil samples with a pH higher than 6.5 but with negligible H^+^ ions [70] by using the following formulas:

ECEC_(pHKCl < 6.5)_ (cmol_(+)_ kg^−1^) = ∑(exchangeable K^+^ + Ca^2+^ + Mg^2+^ + Na^+^ + Mn^2+^ + Fe^2+^ + Al^3+^ + H^+^) (1)

ECEC_(pHKCl > 6.5)_ (cmol_(+)_ kg^−1^) = ∑(exchangeable K^+^ + Ca^2+^ + Mg^2+^ + Na^+^ + Mn^2+^ + Fe^2+^ + Al^3+^) (2)

### 2.3. Soil Amendment Treatments and Research Design for the Small-Scale Experiment Setup

The influence of seven different soil amendment treatments on the total soil Cd and bioavailable Cd concentrations and total Cd accumulation in rice grains (rice variety: Yixiangyou 2115) was tested. The dosages m^−2^ in each of the seven amendments were designed following recommendations by Li and Xu [44] and Hamid et al. [71]. The dosages and treatment codes are shown in Table 1 below.

The chemical formula of rice straw biochar was not determined, but please refer to [72].

Soil amendment treatment implementation started on 10 January 2017 by first broadcasting the respective amendment treatments on the soil surface, followed by shallow plowing (10–15 cm deep) in 2017. Plants grew under soil amendments for six months before harvesting. After this time, manual harvesting of all the rice grains was done to calculate the yield per hectare. A 1 kg sample from the harvested rice grains was kept in a separate self-sealing polyethylene bag and to be later used for elemental analyses. The pretreatment of soil and grain samples was the same as the previous description.

A randomized block design with five replicates for each treatment and a total of 35 blocks [(6 treatments + 1 control) × 5 replicates] was used for the experiment. Each block was 20 m^2^ (5 m × 4 m), surrounded by a 40 cm high wall, insulated with plastic from the bottom (1.0 m) of the soil layer, with 1 m buffer strips to exclude the neighboring treatment effects (Figure S1).

#### Rice Cultivation and Management

The irrigation, main fertilization regime, and pest management of all experimental plots were consistent. Rice seedlings were sown on 15–26 March 2017 and transplanted on 20–30 April 2017. All rice fields were irrigated by flooding [rice fields immersed in water (2–5 cm deep) from transplanting to maturity, followed by drainage on 6–11 August 2017, and then harvested on 20–25 August 2017.

Fertilization was done using the broadcasting method before transplanting. All blocks were fertilized with urea (CH_4_N_2_O) 75 kg ha^−1^, potassium sulfate (K_2_SO_4_) 75 kg ha^−1^, and ammonium phosphate [(NH_4_)_3_PO_4_] 150 kg ha^−1^. Pest control was done by spraying with insecticides (Chlorpyrifos: 1.5 L ha^−1^ + 10% flubendiamide: 300 g ha^−1^) to control *Chilo suppressalis* (rice stem borer) and *Lissorhoptrus oryzophilus* (rice water weevil) once in May 2017.

### 2.4. Implementation of Different Soil Amendment Treatments for Large-Scale Farm Experiment Setup

After obtaining results from the previous experiments testing the effects of the different soil amendment treatments on rice, validation of the effectiveness of combining different Cd remediation techniques into larger-scale farm setups (12.5 hectares) that include different fertilization and plowing schemes was done. As will be explained in the results section in more detail, based on the initial small-scale experiment, it was observed that adding humic acid and silicon fertilizer yielded good results among the soil fertilization treatments. After selecting the treatments that led to (i) lower rhizosphere soil-bioavailable Cd concentrations, (ii) higher grain yield, and (iii) lower Cd accumulation in grains relative to the control treatment (T7). Two fertilizer types were selected i) a silicate fertilizer (sodium metasilicate (Na_2_SiO_3_), 0.03 kg m^−2^ (T2) and humic acid, 0.07 kg m^−2^ (T3).

Consequently, here, rice plants were grown under humic acid treatment, and then compared the effects on both Cd stabilization and accumulation in rice grains under different field managements: sole soil dressing silicon fertilization, soil dressing silicon fertilization supplemented with foliar dressing silicon fertilization, and different plowing depths to determine if further decreases in rice Cd could be obtained.

#### Combination Treatments and Research Design for the Large-Scale Farm Experiment Setup

Here, five different combination treatments were designed, as shown in Table 2 below.

To implement the below-designed combination treatments, five towns were selected in Jinyang county. Each town was considered an experimental site, and on each experimental site, the five designed treatments were applied. Hence 25 plots across the five towns of Jinyang county. Each experimental site (250 m × 250 m) field was divided into five 0.5-hectare blocks (one block for each combination treatment, including the control). Each block was surrounded by a 40 cm high wall, insulated with plastic from the bottom (1.0 m deep). A 1 m buffer strip was left between plots to exclude the neighboring effects among blocks (Figure S1).

Humic acid (lignite derived) and silicon fertilizers (natural wollastonite derived) were purchased from the Chengdu Huayao chemical company. Rice cultivation and management

The irrigation, main fertilization regime, and pest management of all experimental plots were consistent. Rice seedlings were sown on 15–26 March 2018 and transplanted on 20–30 April 2018. All rice fields were irrigated by flooding [rice fields immersed in water (2–5 cm deep) from transplanting to maturity, followed by drainage on 6–11 August 2018, and then harvested on 20–25 August 2018.

Fertilization was done using the broadcasting method before transplanting. All blocks were fertilized with urea (CH₄N₂O) 75 kg ha^−1^, potassium sulfate (K_2_SO_4_) 75 kg ha^−1^, and ammonium phosphate [(NH_4_)_3_PO_4_] 150 kg ha^−1^. Pest control was done by spraying with insecticides (Chlorpyrifos: 1.5 L ha^−1^ + 10% flubendiamide: 300 g ha^−1^) to control *Chilo suppressalis* (rice stem borer) and *Lissorhoptrus oryzophilus* (rice water weevil) once in May 2018.

### 2.5. Preparation of Samples for Analysis

After collection, the soil samples were immediately put into a self-sealing polyethylene bag and transferred to the laboratory. Once in the laboratory, the soil was air-dried to constant weight at room temperature under a shade for ten days; coarse debris was removed by sieving the soil through a 2 mm nylon sieve. The soil was then crushed with an agate pestle and mortar, homogenized with a 70 µm sieve, put in paper envelopes, and stored in a dry cupboard until further analyses. Rice grains were cleaned off the debris (straw), air dried under a shade to constant weight, put in ziplock polythene bags, and then again stored in a dry cupboard.

### 2.6. Determination of Seed Germination Index (SGI)

The SGI was calculated following a method described by Cao et al. [73]. In short, the number of germinated seeds was counted ten days after planting. *GI* = ∑*Gt*/*t*, where *Gt* is the number of germinated seeds on day *t*. Based on the number of germinated seeds, the germination index (*GI* = ∑(*G_t_*/*T_t_*), where *G_t_* is the number of the germinated seeds on Day *t*, and *T_t_* is the time corresponding to *G_t_* in days).

### 2.7. Determination of Cd Concentrations in Soil and Grain Samples

Soil samples were digested with HNO_3_-HClO_4_-HF (v/v/v: 5:1:1) for analysis of total Cd contents using the Kjeldahl digesting block. Soil-bioavailable Cd was extracted with diethylene triamine pentaacetic acid (DTPA) extracting agent (0.005 mol L^−1^ DTPA, 0.01 mol L^−1^ CaCl_2_, and 0.1 mol L^−1^ triethanolamine, pH 7.3). Rice grain samples were rinsed in deionized water, air-dried, de-husked, ground passed through a 40-mesh sieve, and then digested with HNO_3_-HClO_4_ (v/v: 4:1). Total Cd concentration in soil and grain samples was determined by inductively coupled plasma mass spectrometry (ICP-MS, Agilent 7500a, Agilent Technologies Inc, Palo Alto, USA).

### 2.8. Data Analysis

The data were tested by a one-way ANOVA using the SPSS 16.0 for Windows statistical software package (SPSS Inc., Chicago, IL, USA). Before ANOVAs, the data were checked for normality and the homogeneity of variances and log-transformed to correct deviations from these assumptions when needed. The analyses were performed with the general linear ANOVA model procedure. Post-hoc comparisons were tested using Tukey’s test at a significance level of *p* < 0.05. The significance of differences between the treatment groups and control group were evaluated by Tukey’s HSD. Likewise, the soil or grain Cd concentrations were compared with the corresponding national standards to determine whether they were lower than the corresponding safety values after remediation. Figures were made using Origin 2019b (Origin Lab., Northampton, MA, USA).

## 3. Results

### 3.1. Cd Concentration in the Surrounding Environment

**Figure 1:**
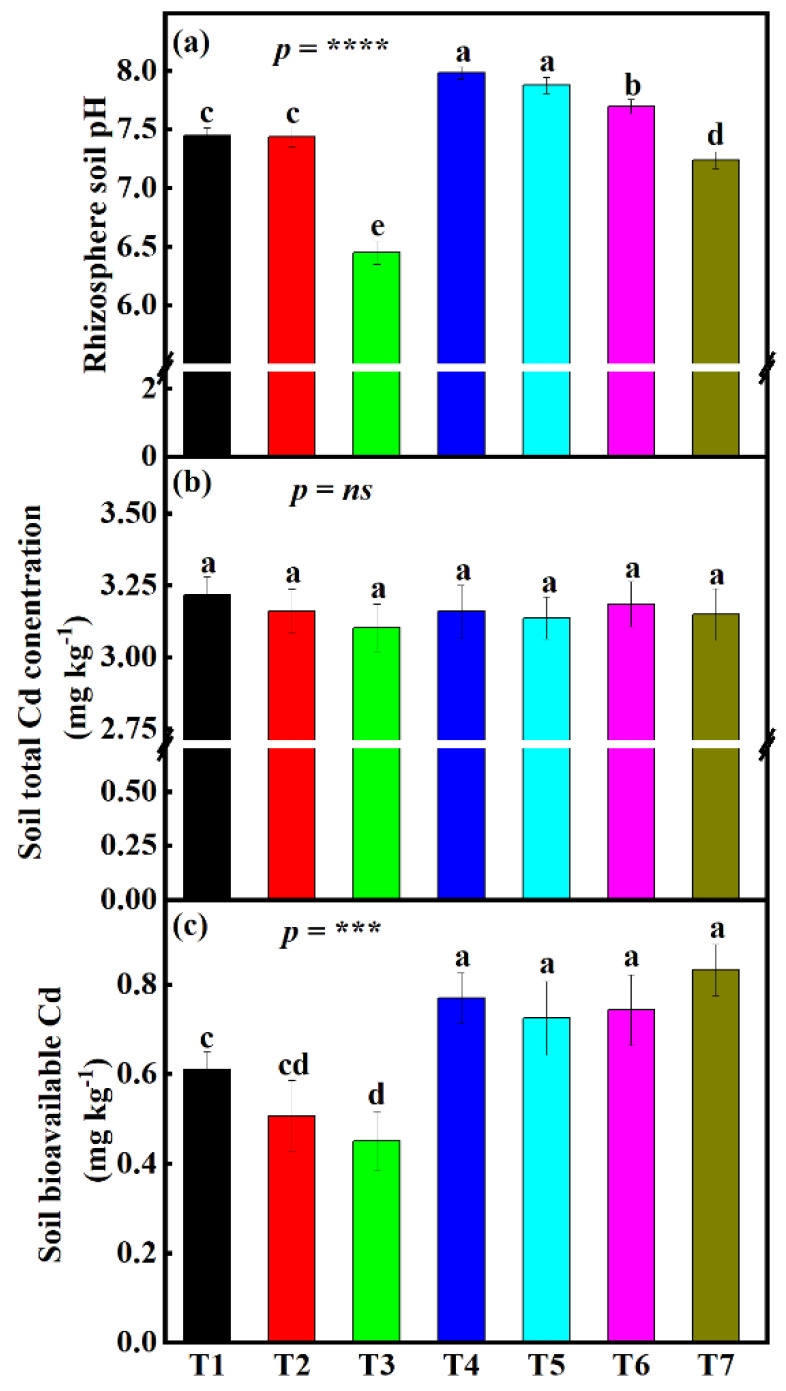
Influence of different fertilizer treatments on rhizosphere (**a**) rhizosphere soil pH, (**b**) soil total Cd concentrations, and (**c**) soil bioavailable Cd under small-scale plot experiment. Data (means ± SE, n = 5) followed by different subscripts denotes significant differences between treatments at *p* < 0.05 according to Tukey’s HSD. *p*-values of one-way ANOVA, are indicated: *p* < 0.001, ***; *p* < 0.0001, ****; *ns*, not significant. Treatments: sepiolite (Mg_4_Si_6_O_15_(OH)_2_•6(H_2_O)), 1.27 kg m^−2^ (T1); sodium metasilicate (Na_2_SiO_3_), 0.03 kg m^−2^ (T2); humic acid, 0.07 kg m^−2^ (T3); calcium magnesium phosphate fertilizer, 0.09 kg m^−2^ (T4); ferrous sulfate (FeSO_4_), 0.07 kg m^−2^ + manganese sulfate (MgSO_4_), 0.007 kg m^−2^ (T5); 6) straw biochar, 0.75 kg m^−2^ (T6); and the control treatment (no chemicals added) (T7).

To understand the source of Cd contamination in the experimental area, samples from (i) different soil depths (Figure S2), (ii) irrigation water (river) (Figure S3), and (iii) walls of a 100-year-old building (Figure S4) were analyzed. Experimental results showed that the Cd concentration from the irrigation water was 0.0091 mg L^−1^, within the FAO safety level (0.01 mg L^−1^) (Table 3). It was also found that Cd concentrations increased (ranging from 3.63–4.43 mg kg^−1^) with soil depth (20–140 cm). Intriguingly, results from the >100-year-old clay building showed that Cd concentration levels were 3.85 mg kg^−1^ (Figure 1).

### 3.2. Influence Different Fertilizer Treatments on Rhizosphere pH Change under the Small-Scale Plot Experiment

The results from the current experiment showed that the seven different amendment treatments used had a significant influence (*p* < 0.0001) on rhizosphere pH (Figure 1a). Treatment T3 had the lowest pH, while T4 had the highest pH. To be specific, treatments T2 and T3 led to a significant decline in pH (5.7%, and 10.9%, respectively) relative to treatment T7, with 7.24. There was also a significant difference in measured pH between T2 and T3. Furthermore, it was also found that treatments T1, T4, T5, and T6 had led to significant pH increases of 2.7, 9.3, 8.1, and 6%, respectively, relative to treatment T7. Insignificant differences were only found between treatments T1, T2, T4, and T5.

### 3.3. Influence of Different Fertilizer Treatments on Rhizosphere Soil-Available Cd and Soil Total Cd Concentrations under Small-Scale Plot Experiment

Experiment results showed a significant (*p* < 0.001) change in the bioavailable Cd concentrations after the application of the 7 amendment treatments (Figure 1c). It was found that there was a decline in soil bioavailable Cd concentrations T1 (26.8%), T2 (39.2%), T3 (7.6%), T4 (7.6%), T5 (13%), T6 (10.8%), and T7 (30.3%) compared to C treatment. However, only T1, T2, and T3 were significantly different from treatment T7. Moreover, there was no significant difference between treatment T1 and T2 and treatment T2 and T3. Furthermore, there was no significant difference amongst T4, T5, T6, and T7.

For soil total Cd, an insignificant increase (*p* = *ns*) in total Cd concentrations across all the treatments was noticed (Figure 1b). Total Cd concentrations under T1, T2, T3, T4, T5, T6 ranged between 3.1 and 3.19 mg kg^−1^ while the treatment T7 had 3.15 mg kg^−1^. Soil total Cd concentrations under all treatments far exceeded the national safety standard of GB 15618-2018 in the People’s Republic of China (0.6 mg kg^−1^, the 2nd-grade standard).

### 3.4. Influence of the Different Fertilizer Treatments on Rice Seed Germination Index (SGI), Grain Yield, and Grain Cd Concentration under the Small-Scale Plot Experiment

**Figure 2:**
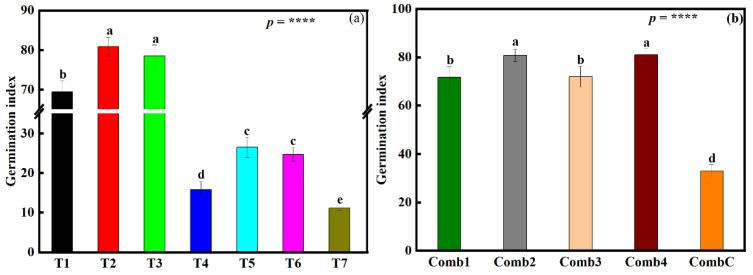
Influence of the different fertilizer treatments on rice seed germination index (SGI) of (**a**) rice seeds sown under the small-scale plot experiment and (**b**) rice seeds sown under the large-scale field experiment. Data (means ± SE, n = 5) followed by different subscripts denotes significant differences between treatments at *p* < 0.05 according to Tukey’s HSD. *p*-values of one-way ANOVA, are indicated: *p* < 0.0001, ****. Treatments for (**a**): sepiolite (Mg_4_Si_6_O_15_(OH)_2_•6(H_2_O)), 1.27 kg m^−2^ (T1); sodium metasilicate (Na_2_SiO_3_), 0.03 kg m^−2^ (T2); humic acid, 0.07 kg m^−2^ (T3); calcium magnesium phosphate fertilizer, 0.09 kg m^−2^ (T4); ferrous sulfate (FeSO_4_), 0.07 kg m^−2^ + manganese sulfate (MgSO_4_), 0.007 kg m^−2^ (T5); 6) straw biochar, 0.75 kg m^−2^ (T6); and the control treatment (no chemicals added) (T7). Treatments for (**b**): humic acid + soil dressing silicon fertilization + deep-plowing (15–30 cm) (Comb1); humic acid + soil silicon dressing fertilization + shallow plowing (5–10 cm deep) (Comb2); humic acid + soil dressing silicon fertilization + deep plowing (15–30 cm) (Comb3); humic acid + soil dressing silicate fertilization + foliar dressing silicon fertilization + shallow plowing (5–10 cm) (Comb4), and; normal plowing with no additional fertilization (CombC).

The ANOVA results showed a significant difference (*p* < 0.0001) in different fertilizers’ influence on rice SGI across all treatments (Figure 2a). Plants under treatment T2 had the highest SGI (86.2%) compared to treatment T7. This was followed by T3, T1, T5, T6, and then lastly T4 (85.8%, 83.9%, 57.9%, 54.8%, and 29.6% respectively). Insignificant differences were noticed between T2 and T3, and T5 and T6).

**Figure 3:**
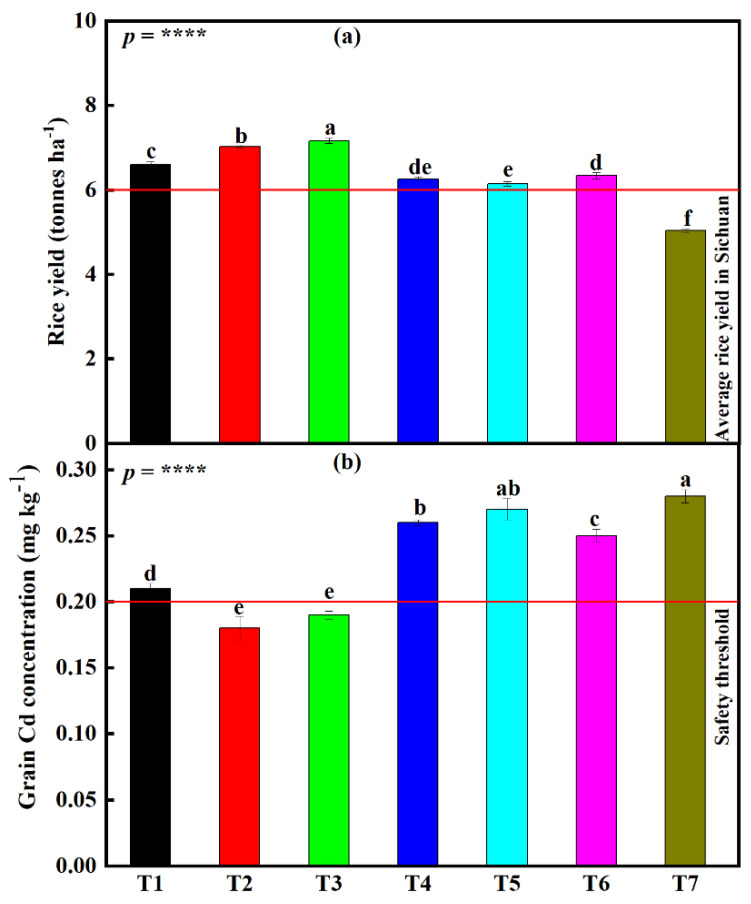
Influence of the different fertilizer treatments on (**a**) rice grain yield and (**b**) rice grain Cd concentration at small-scale plot experiment. Data (means ± SE, n = 5) followed by different letters denote significant differences between treatments at *p* < 0.05 according to Tukey’s HSD. *p*-values of one-way ANOVA, are indicated: ****, *p* < 0.0001. Treatments: sepiolite (Mg_4_Si_6_O_15_(OH)_2_•6(H_2_O)), 1.27 kg m^−2^ (T1); sodium metasilicate (Na_2_SiO_3_), 0.03 kg m^−2^ (T2); humic acid, 0.07 kg m^−2^ (T3); calcium magnesium phosphate fertilizer, 0.09 kg m^−2^ (T4); ferrous sulfate (FeSO_4_), 0.07 kg m^−2^ + manganese sulfate (MgSO_4_), 0.007 kg m^−2^ (T5); 6) straw biochar, 0.75 kg m^−2^ (T6); and the control treatment (no chemicals added) (T7).

A significant difference (*p* < 0.0001) in rice grain yield was also noticed on plants under all seven different fertilization treatments (Figure 3a). From highest to lowest, the rice yield was 7.16 (T3), 7.02 (T2), 6.6 (T1), 6.34 (T6), 6.26 (T4), 6.14 (T5), and 5.03 (T7). Insignificant differences were also noticed between treatments, T4 and T5 and also between treatments T4 and T6 were noticed. It is also worth mentioning that only the yield under treatment T7 did not surpass the average rice grain yield in Sichuan province, China (6 tonnes ha^−1^) (Figure 3a).

When measuring Cd accumulation in rice grains, a significant difference (*p* < 0.0001) was found amongst the different fertilizer treatments (Figure 3b). Cd accumulation in rice grains was highest under treatment T7 (0.28 mg kg^−1^). From highest to lowest, the use of different amendment treatments led to a 47.4, 35.7, 25, 10.7, 7.7, and 3.6% decline in Cd accumulation in rice grains, T3, T2, T1, T6, T4, and T5, respectively. An insignificant difference was noticed between treatments T2 and T5. However, it is also worth noting that only treatment T2 (0.18 mg kg^−1^) and T3 (0.19 mg kg^−1^) managed to produce grains with a Cd concentration lower than the safety threshold (0.2 mg kg^−1^) (Figure 3b).

### 3.5. Effects of Different Soil Amendment Combinations on Rhizosphere pH under the Large-Scale Field Experiment

**Figure 4:**
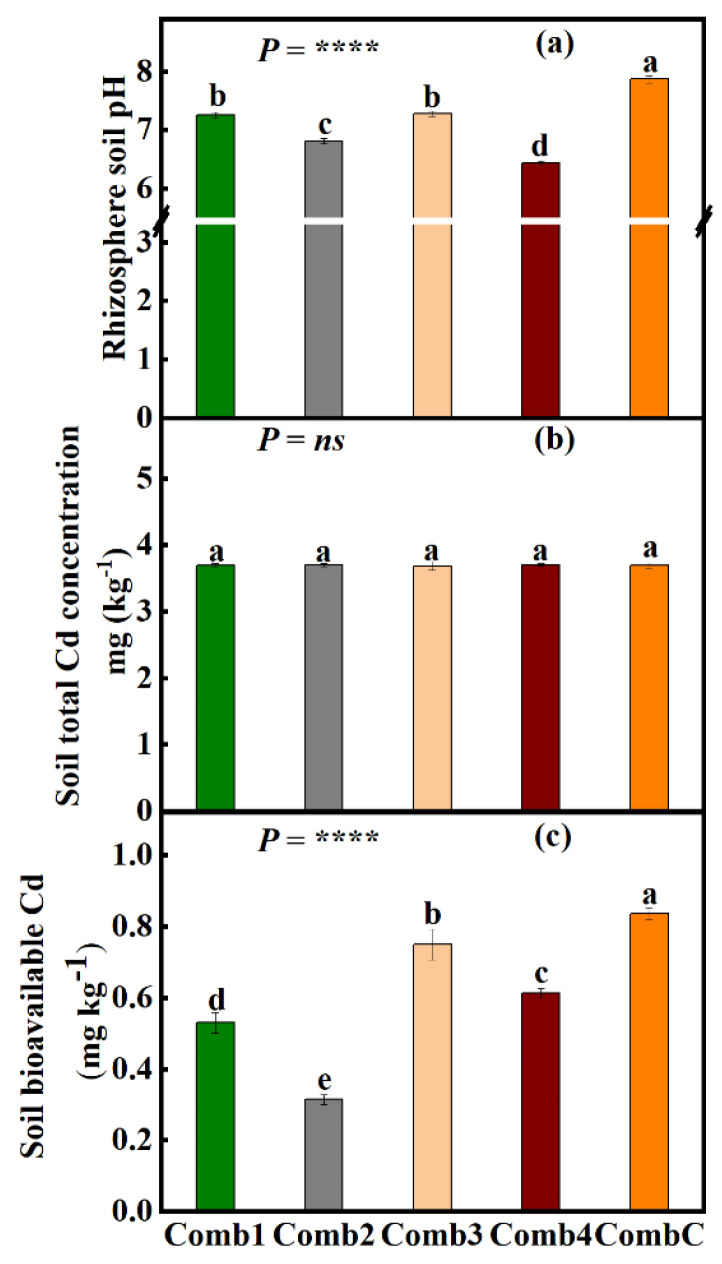
Effects of different combinations treatments on rhizosphere (**a**) soil pH, (**b**) soil total Cd concentrations, and (**c**) soil-bioavailable Cd at large field scale. Data (means ± SE, n = 5) followed by different subscripts denotes significant differences between combinations of treatments at *p* < 0.05 according to Tukey’s HSD. *p*-values of one-way ANOVA, are indicated: ****, *p* < 0.0001; *ns*, not significant. *Combination treatments*: humic acid + soil dressing silicon fertilization + deep-plowing (15–30 cm) (Comb1); humic acid + soil silicon dressing fertilization + shallow plowing (5–10 cm deep) (Comb2); humic acid + soil dressing silicon fertilization + deep plowing (15–30 cm) (Comb3); humic acid + soil dressing silicate fertilization + foliar dressing silicon fertilization + shallow plowing (5–10 cm) (Comb4), and; normal plowing with no additional fertilization (CombC).

After implementing different fertilizer combinations and plowing depths, a significant difference (*p* < 0.001) in rhizosphere soil pH change was observed across amendment combinations (Figure 4a). Amendment Comb4 had the highest percentage change (decline) in pH (18.3%) compared to CombC, followed by Comb2 and Comb1 (13.5% and 7.8% respectively), then lastly, Comb3 (7.5%). It was also found that an insignificant difference in rhizosphere pH existed between combination treatments Comb3 and Comb4.

### 3.6. Effects of Different Soil Amendment Combinations on Rhizosphere Soil Total Cd Concentrations and Bioavailable Cd under the Large-Scale Field Experiment

Contrary to pH change, total soil Cd concentration was insignificantly influenced (*p* < *ns*) by the use of different fertilizer amendments and plowing depths (Figure 4b). The total Cd concentration was 3.7 mg kg^−1^ across all fertilizer amendment treatments. Soil total Cd concentrations under all combination treatments far exceeded the national safety standard of GB 15618-2018 in the People’s Republic of China (0.6 mg kg^−1^, the 2nd-grade standard).

Although the differences in total Cd concentrations among different combinations were insignificant (Figure 4c), soil-bioavailable Cd was significantly influenced by the different amendment combinations (*p* < 0.0001) (Figure 4b). It was also found that soil under amendment combination Comb2 had the highest decline in bioavailable Cd concentrations (62.3%), followed by Comb1 (36.6%), then Comb4 (26.6%), and last Comb3 (10.3%) relative to CombC. All the amendment combination treatments were significantly different from each other.

### 3.7. Effects of Different Combination Treatments on Rice Growth and Yield Parameters under the Large-Scale Field Experiment

#### 3.7.1. Rice Seed Germination Index (SGI), Panicle Length (PL), Number of Tillers per Hill (NTH), and Number of Kernels per Panicle (NKP) at Large Field Scale

The ANOVA results showed that SGI was significantly influenced by combination treatment (*p* < 0.0001) (Figure 2b). SGI increased by 146.2, 145.5, 118.6, and 117.8% (Comb4, Comb2, Comb3, and Comb1, respectively) compared to CombC. An insignificant difference between combination Comb1 and Comb3 and also between Comb2 and Comb4 was also noticed.

It was also noticed that there was a significant difference (*p* < 0.0001) in the NTH across different combination treatments (Table 4). The NTH increased by 67.5, 62.1, 38.9, and 20.6% (Comb4, Comb3, Comb2, and Comb1, respectively) relative to the control (CombC). All the different combination treatments were significantly different from each other.

The results from the experiment showed a significant difference in the rice PL across all combination treatments (Table 4). The results followed the same trend as NTH. The PL ranged from 8.3 to 14.2 cm. All combination treatments led to an increase in PL relative to CombC. PL increased by 71.1, 66.3, 37.3, and 24.1% (Comb4, Comb3, Comb2, and Comb1, respectively) compared to CombC. An insignificant difference between Comb3 and Comb4 was also noticed.

The experiment results also showed that NKP was significantly different (*p* < 0.0001) across all combination treatments (Table 4). NKP ranged from 28.4 to 44.6. Compared to the control treatment, NKP increased by 57, 41.5, 25, and 16.5% (Comb4, Comb3, Comb2, and Comb1, respectively). NKP values under all combination treatments were significantly different from each other.

#### 3.7.2. Differences in Rice Grain Yield and Grain Cd Concentration under the Large-Scale Field Experiment

**Figure 5:**
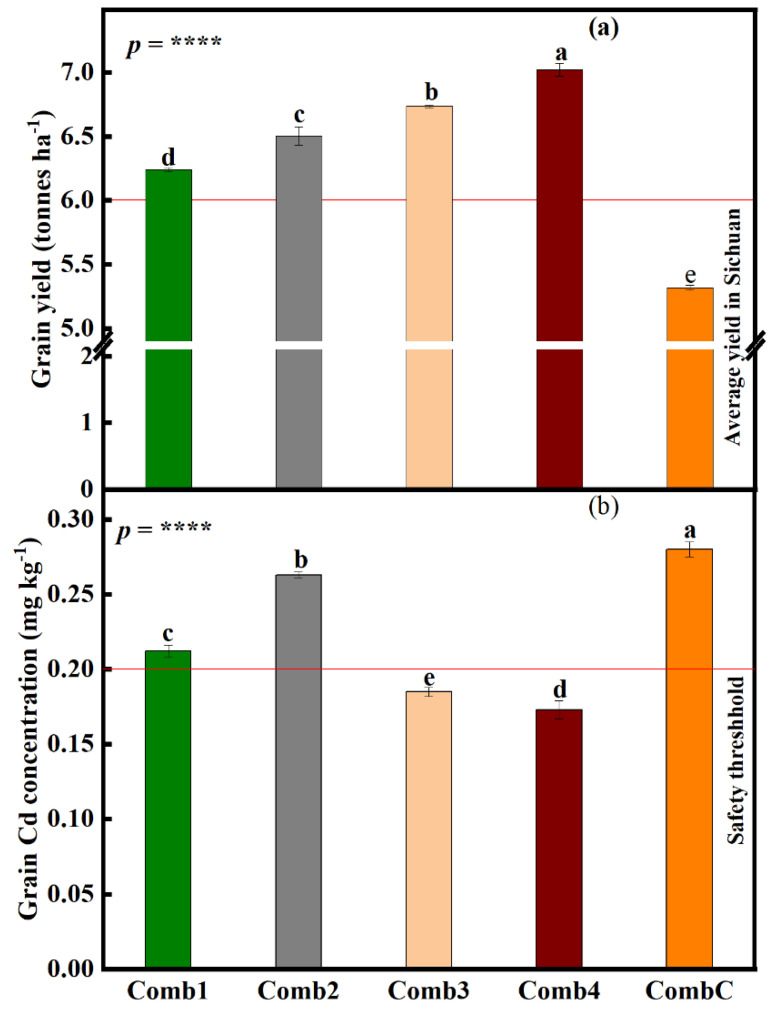
Effects of different combination treatments on (**a**) grain yield and (**b**) grain Cd concentrations under the large-scale field experiment. Data (means ± SE, n = 5) followed by different subscripts denotes significant differences between treatments at *p* < 0.05 according to Tukey’s HSD. *p*-values of one-way ANOVA, are indicated ****, *p* < 0.0001. Combinations treatments: humic acid + soil dressing silicon fertilization + deep-plowing (15–30 cm) (Comb1); humic acid + soil silicon dressing fertilization + shallow plowing (5–10 cm deep) (Comb2); humic acid + soil dressing silicon fertilization + deep plowing (15–30 cm) (Comb3); humic acid + soil dressing silicate fertilization + foliar dressing silicon fertilization + shallow plowing (5–10 cm) (Comb4), and; normal plowing with no additional fertilization (CombC).

The experiment results showed that rice grain yield was significantly influenced by combination treatments (*p* < 0.0001) (Figure 5a). Relative to CombC, which had the lowest yield (5.3 tonnes ha^−1^), grain yield significantly increased by 14.9, 10.3, 6.4, and 2.1% (Comb4, Comb3, Comb2, and Comb1, respectively). All treatments were significantly different from each other. It is also worth noting that yields under all combination treatments were higher than the average rice yield in Sichuan province (6 tonnes ha^−1^).

The Cd concentrations in rice grains were also checked, and found that Cd concentrations were significantly influenced by combination treatments (*p* < 0.0001) (Figure 5b). Rice grains under CombC treatment had the highest Cd concentration (0.28 mg kg^−1^), which was slightly higher than the safety threshold level in China (0.2 mg kg^−1^). Compared to CombC treatment, grain Cd concentration declined by 32.2, 30.4, 24.3, and 6.1% (Comb4, Comb3, Comb1, and Comb2, respectively). Although grain Cd concentrations under all combination treatments were significantly different from each other, only concentrations under Comb3 and Comb4 managed to go below the safety threshold.

### 3.8. Correlations between Cd Soil and Plant Attributes under the Large Field Scale

**Figure 6:**
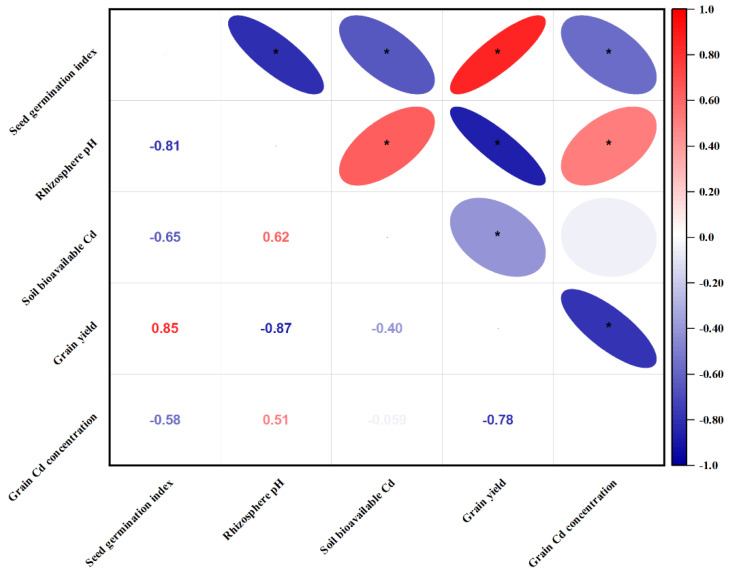
Correlation matrix showing relationships between seed germination index, rhizosphere pH, soil bioavailable Cd, grain yield, and grain Cd concentration under a large-scale field experiment. Red color denotes positive relationships; blue color denotes negative relationships. Left-inclined ellipses denote a positive relationship, right inclined ellipses denote a negative relationship. Asterisks represents significant correlations.

A Pearson’s correlation analysis was performed (Figure 6), and the results showed a very strong negative relationship (*r* = −0.81) between rhizosphere soil pH and SGI, a very strong negative relationship (*r* = −0.65) between bioavailable soil Cd and SGI, and also a very strong positive relationship (*r* = 0.85) between SGI and grain yield. Rhizosphere soil pH also had a strong positive relationship (*r* = 0.51) with grain Cd concentration, a very strong negative relationship (*r* = −0.87) with grain yield, and a strong positive relationship with bioavailable soil Cd. A very strong negative relationship (*r* = −0.78) between grain yield and grain Cd concentration was also noticed. Soil bioavailable Cd concentration also had a strong negative relationship (*r* = −0.4) with grain yield. However, intriguingly, the relationship between soil available Cd and grain Cd concentration was insignificant.

## 4. Discussion

Cd contamination on farmland is a global problem leading to massive declines in yield and human health hazards [74]. In this experiment, it was observed that soil amendment with different fertilizer combinations and different plowing depths partly improves rice growth and yield quality. That is, using a combination of soil fertilizer, foliar fertilizer, and shallow plowing shows lower soil bioavailable Cd, higher rice growth and yield, and lower Cd concentration in grains. The underlying mechanism is, however, intricate as it includes lower Cd uptake and improving SGI, NTH, PL, and NKP. To the best of the knowledge of this paper’s authors, this current study is the first to provide a comprehensive assessment of the effects of amalgamated different soil amendments material and tillage practices on rice production in Cd-contaminated paddies in the Sichuan province.

### 4.1. Humic Acid Effectively Lowers Rhizosphere Soil pH

Different soil amendments are usually used for the remediation of heavy metal-contaminated soils. They are thought to be effective by reducing the soil’s available heavy metals through increases in the soil pH, altering the chemical form of heavy metal, restraining the heavy metal in the soil as well as enhancing the soil microbial activities [75,76,77,78,79]. On the one hand, in the small-scale plot experiment, significant effects of humic acid in lowering rhizosphere pH were observed (Figure 1a). This result was corroborated by researchers such as Ali and Mindari [80] and Tan et al. [81], who reported the effectiveness and efficiency of humic acid in lowering soil pH. On the other hand, in the large-scale field experiment, rhizosphere pH was significantly lowered by all amendment combinations except for CombC, which was the control treatment (Figure 4a). This might be due to the presence of humic acid.

### 4.2. Different Amendment Materials Effectively Lowers Rhizosphere Bioavailable Cd

Although none of the treatments, both under the small-scale plot experiment and the large-scale field experiment, managed to lower the total soil Cd concentrations (Figure 1c and Figure 4c), some significantly reduced the Cd bioavailability. For instance, in the preliminary plot experiment, bioavailable Cd was significantly lowered in the rhizospheres under treatment T1, T2, and T3. This is because (i) treatment T1 contains sepiolite, and sepiolite as an absorbent has been reported to stabilize heavy metals, including Cd [52,82], which may also contribute to the reduction of Cd bioavailability [83]. (ii) treatment T2 contains sodium metasilicate. Several studies have reported that Si also immobilizes Cd in soil [84], hence the reduction of Cd bioavailability under T2. (iii) treatment T3 contains humic acid. Humic acid has also been reported to reduce the bioavailability of heavy metals by strong affinity and ability to form a stable chelate with metal ions, with carboxylic groups and phenolic-OH being the dominant binding groups in HA; this effect varies by metals [85,86]. Metals have different affinities for humic acid based on their stability constants, for instance, for Cd (7.8), Cu (13.3), Pb (14.80), and Zn (8.1) [87]. The influence of humic acid and sodium metasilicate mentioned above also explains the decline in Cd bioavailability in soils under the large-scale field experiment (Figure 4c). Furthermore, these results of low Cd availability in the large-scale experiment may be due to low pH values under the same treatment (Figure 4a). The pH value plays an essential role in the bioavailability and mobility of heavy metals in soil [88]. This is also corroborated by the correlation results (Figure 6), where it was found a strong positive relationship between rhizosphere pH and soil bioavailable Cd. It is therefore suggested that more research be done to elucidate the effect of humic acid and sodium metasilicate on subcellular Cd distribution in rice tissues, including the grains, and also on the distribution of different Cd chemical forms.

### 4.3. Soil Amendment Treatments Improve Seedling Germination and Plant Growth

Seedling germination is one of the crucial stages in a plant’s life cycle; the higher the population of seedlings that survives this stage, the higher the plant population is in the field. It has been reported that heavy metals in higher concentrations reduce seed germination rates, growth, and processes mainly associated with the physiological, biochemical, and genetic elements of the plant system [9,89]. In the small-scale experiment, SGI was significantly higher under T1, T2, and T3. These treatments have been described above to contain a chemical that stabilizes Cd and reduces their toxicity to plants, while T1 contains sepiolite, which has also been reported to stabilize and reduce Cd bioavailability. It is also worth mentioning that the effects of T2 and T3 are the same reason why higher SGI values are being found in the amended treatments Comb1, Comb2, Comb3, and Comb4 relative to the CombC during the large-scale field experiment regardless of the plowing depth (Figure 2b). Furthermore, the even higher SGI values under combination treatments that included shallow plowing depth might be because when soil total Cd concentrations were at different soil depths determined (Figure 4b), it was found that Cd concentration increased with soil depth (Figure S5). When the Cd concentration from the walls of a >100-year-old building was determined (Table 3 and Figure S4) to develop an idea of past soil Cd concentrations when the building was built, it was also found that Cd concentrations were even higher than those currently found in the field. When the samples of the irrigation water (Figure S3) were analyzed, it was also found that their Cd concentration was low (0.0091 mg L^−1^) (Table 3). This led to the conclusion that the source of Cd in farmland (experimental area) was the parent rock. Under normal circumstances, the top rhizosphere soil layer contains lesser Cd concentrations because most of it gets absorbed by plants with time; also, some of it gets leached away. If the fields are deep plowed, this leads to the exhumation of soil with an even higher Cd concentration and introducing it to the top surface layer exposing it to the seed, hence more bioavailable Cd. This increases Cd toxicity under deep plowing treatment and hence more Cd toxicity to plants leading to a lower SGI under deep plowed treatments relative to shallow plowed (Figure 2b). These results are also supported by the correlation results (Figure 6), which showed a very strong negative relationship between Cd bioavailability and SGI. It is therefore suggested that more research be done to understand the influence of these treatments and management practices on SGI over a long-term period.

In plants, Cd toxicity varies with the experimental settings and growth conditions and depends on the availability of Cd, exposure time, and the plant growth stages [90,91]. The disparities in Cd bioavailability under different soil amendment treatments in the large-scale experiment may also attribute to different growth characteristics reported in (Table 4). The higher the bioavailable Cd, the higher the toxicity to plants, and the lower the number of NTH, PL, and NKP. Various studies have been documented reporting the influence of heavy metals on rice tiller number [92,93], PL [94,95], and NKP [96]. Furthermore, in the large-scale experiment, it was also noticed that these growth attributes were higher under shallow relative to deep plowing amendments (Table 4). Even-higher values were noticed under amendment treatments that included soil and foliar Si fertilizers. Rice is a Si-loving plant, and Si is responsible for stimulating rice growth and boosting antioxidant activity in rice to build tolerance against abiotic stress [97,98,99]. Also, foliar fertilizers are effectively absorbed by plants due to less competition for absorption sites with other mineral ions compared to soil fertilizer-dressed fertilizers [100,101].

### 4.4. Different Combination Treatments Variedly Improve Grain Yield and Grain Cd Concentration

Heavy metals, once deposited beyond certain permissible limits, obnoxiously influence the density, composition, and physiological activities of microbiota, dynamics, and fertility of the soil, leading eventually to a decline in crop production and via the food chain, and human and animal health [102]. This decline in production also includes a reduction in grain yield and yield quality. The highest grain yield was under Comb4 (humic acid + soil silicate fertilizer + foliar silicate + shallow plowing). The reason for this might be that: (i) as explained before, deep plowing increased Cd concentrations, hence higher toxicity. This means lower Cd toxicity in shallow-plowed fields and higher rice growth leading to higher grain yield. (ii) Since the soil growing conditions (pH and Cd bioavailability) have been improved by the addition of humic acid and silicone fertilizer, as explained before, this led to higher SGI, NTH, PL, and NKP, hence leading to a higher grain yield. (iii) The foliar application of the silicone fertilizer improved plant absorption of Si through the leaves, hence improving plants’ tolerance to Cd toxicity and growth and yield. Silicon fertilizers have been extensively applied in rice fields for increasing productivity, and these results are supported by several authors who reported enhancement of rice grain yield through the application of foliar silicon fertilizer [103,104]. These explanations are consistent with the correlation results (Figure 6), where a very strong negative relationship between grain yield and rhizosphere pH was found, and a strong negative relationship between grain yield and soil bioavailable Cd was also found. Rice yield involves a long-term, continuous method. Through this method, plants in nature rely mostly on their ability to resist the toxic effect of Cd; it is therefore suggested that more research be conducted to understand the long-term influence of these amendment approaches on rice grain yield under alkaline soils. 

The main aim of this experiment was to produce rice grains with a low Cd concentration. Based on this experiment results, it is found that grain Cd concentrations were lowest under treatment Comb4 (Figure 5b). This might be attributed to shallow plowing and supplementation of soil silicon fertilizer with foliar silicon fertilizer, as explained before. Furthermore, some studies reported that Si inhibits Cd uptake and accumulation of plant maize root, shoot, and grain [103,105]. The mobility of Cd in the soil, the concentrations of Cd in the root, and the transportation to aboveground organs depend on the concentration of Si and Cd in the soil–plant system [106,107]. It is therefore suggested that more research be conducted to understand the influence of plant population and intercropping of rice with other heavy metal hyperaccumulators on rice grain yield and Cd accumulation.

## 5. Conclusions

This paper studied the influence of different soil amendment approaches on the safer production of rice with high yield and lower (safer Cd concentrations) in alkaline paddy fields. The addition and implementation of different amendment combinations led to different results of rhizosphere Cd bioavailability, grain Cd concentration, and grain yield. Concerning the reduction of bioavailable Cd in the rhizosphere, combination treatment Comb2 led to the highest reduction of bioavailable Cd concentration in the soil. For the rest of the combination treatments, the order was Comb1, Comb4, Comb3, and lastly, CombC (highest to lowest reduction). Concerning grain yield and quality, on the one hand, Comb4 led to the highest production of grain yield, Comb3, Comb2, Comb1, then CombC in their order from highest to lowest. On the other hand, the lowest grain Cd concentrations were found under the combination treatment Comb4, followed by Comb3, Comb1, Comb2, and then CombC with the highest. Humic acid application, soil silicone fertilizer, foliar silicone fertilizer, and shallow plowing gave the best results of the highest yield together with the safest levels of grain Cd concentrations. It is therefore recommended for rice farmers in Jinyang county that (1) to reduce Cd bioavailability in the soil, they should adopt and implement Comb2 in their farming regimes, and (2) to attain higher rice yields with low grain Cd concentrations and low operational costs, farmers should adopt and implement combination treatment Comb4. However, it is worth noting that for the rest of the farmers around the world, it is advised to first understand the source of Cd contamination in your field before implementing any of the result combination treatments reported in this study.

## Figures and Tables

**Table 1:** The name, codes, chemical formulas, and application rates amendments of different amendment materials used in the small-scale field experiment.

Treatment Codes	Amendment Material Names	Chemical Formulas	Application Rates (kg m^−2^)
T1	Sepiolite	Mg_4_Si_6_O_15_(OH)_2_•6(H_2_O)	1.27
T2	Sodium metasilicate	Na_2_SiO_3_	0.03
T3	Humic acid	C_187_H_186_O_89_N_9_S_1_	0.07
T4	calcium magnesium phosphate fertilizer	CaMgO_4_P^+^	0.09
T5	ferrous sulfate + manganese sulfate	FeSO_4_ + MgSO_4_	0.07 + 0.007
T6	Straw biochar	-	0.75
T7/Control	No chemical added	-	-

**Table 2:** The basic physicochemical properties of the soil before the addition of amendments.

Treatment Codes	Soil Amendment Options				
	Humic Acid	Soil Silicon Fertilizer	Foliar Silicon Fertilizer	Deep Plowing (15–30 cm)	Shallow Plowing (5–10 cm)
Comb1	+	+	−	+	−
Comb2	+	+	−	−	+
Comb3	+	+	+	+	−
Comb4	+	+	+	−	+
CombC	−	−	−	−	+

**Table 3:** The basic physicochemical properties of the soil before the addition of amendments.

Chemical Properties	Value (Mean ± Standard Error)
pH	7.63 ± 0.7
CEC (mg kg^−1^)	12.83 ± 0.9
Exchangeable Ca^2+^ (mg kg^−1^)	17.1 ± 4.7
Exchangeable K^+^ (mg kg^−1^)	76.0 ± 21.2
Exchangeable Fe^2+^ (mg kg^−1^)	476.9 ± 24.8
Exchangeable NH_4_^+^ (mg kg^−1^)	28.02 ± 0.01
Exchangeable Na^+^ (%)	0.001 ± 0.0001
Exchangeable Mn^2+^ (mg kg^−1^)	6.1 ± 0.2
Exchangeable Mg^2+^ (mg kg^−1^)	20.4 ± 0.8
Available P (mg kg^−1^)	1.24 ± 0.08
Available NO_3_^−^ (mg kg^−1^)	15.88 ± 0.93
Total soil Cd (mg kg^−1^)	3.21 ± 0.03
Irrigation water (mg L^−1^)	0.0091 ± 0.0001
Total Cd concentration from old clay building walls (mg kg^−1^)	3.8471 ± 0.02

Data (means ± SE, n = 5).

**Table 4:** Effect of different combinations treatments on the growth attributes of rice grown in Cd contaminated soil under large-scale field experiment.

Treatments	Number of Tillers per Hill	Panicle Length (cm)	Number of Kernels per Panicle
Comb1	6.20 ± 0.5_d_	10.3 ± 0.1_c_	33.1 ± 0.9_d_
Comb2	7.14 ± 0.4_c_	11.4 ± 0.3_b_	35.5 ± 7_c_
Comb3	8.33 ± 0.5_b_	13.8 ± 0.6_a_	40.2 ± 1_b_
Comb4	8.61 ± 0.8_a_	14.2 ± 0.3_a_	44.6 ± 1.4_a_
CombC	5.14 ± 0.4_e_	8.3 ± 0.4_d_	28.4 ± 2.1_e_
*p*-value	****	****	****

Data (means ± SE, n = 5) followed by different subscripts in the same column denotes significant differences between combination treatments at *p* < 0.05 according to Tukey’s HSD. *p*-values of one-way ANOVA, are indicated ****, *p* < 0.0001. *Combinations treatments*: humic acid + soil dressing silicon fertilization + deep-plowing (15–30 cm) (Comb1); humic acid + soil silicon dressing fertilization + shallow plowing (5–10 cm deep) (Comb2); humic acid + soil dressing silicon fertilization + deep plowing (15–30 cm) (Comb3); humic acid + soil dressing silicate fertilization + foliar dressing silicon fertilization + shallow plowing (5–10 cm) (Comb4), and; normal plowing with no additional fertilization (CombC).

## Data Availability

No new data were created or analyzed in this study. Data sharing is not applicable to this article.

## Supplementary Materials

The following supporting information can be downloaded at: https://www.mdpi.com/article/10.3390/foods12020314/s1, Figure S1: Experimental field, with different amendments; Figure S2: Sampling of Cd-contaminated soil from different layers of soil; Figure S3: Irrigation water sampling from different sources; Figure S4: The sampling of wall mud, from a 100-year-old building; Figure S5: The soil depth and Cd concentration.
