# Supplementary material for: Reduction of Cd Uptake in Rice (Oryza sativa) Grain Using Different Field Management Practices in Alkaline Soils
Source: Foods. 2023 Jan 9;12(2):314. doi: 10.3390/foods12020314 (PMC9858237; doi:10.3390/foods12020314)

Figure S1. Experimental field, with different amendments.

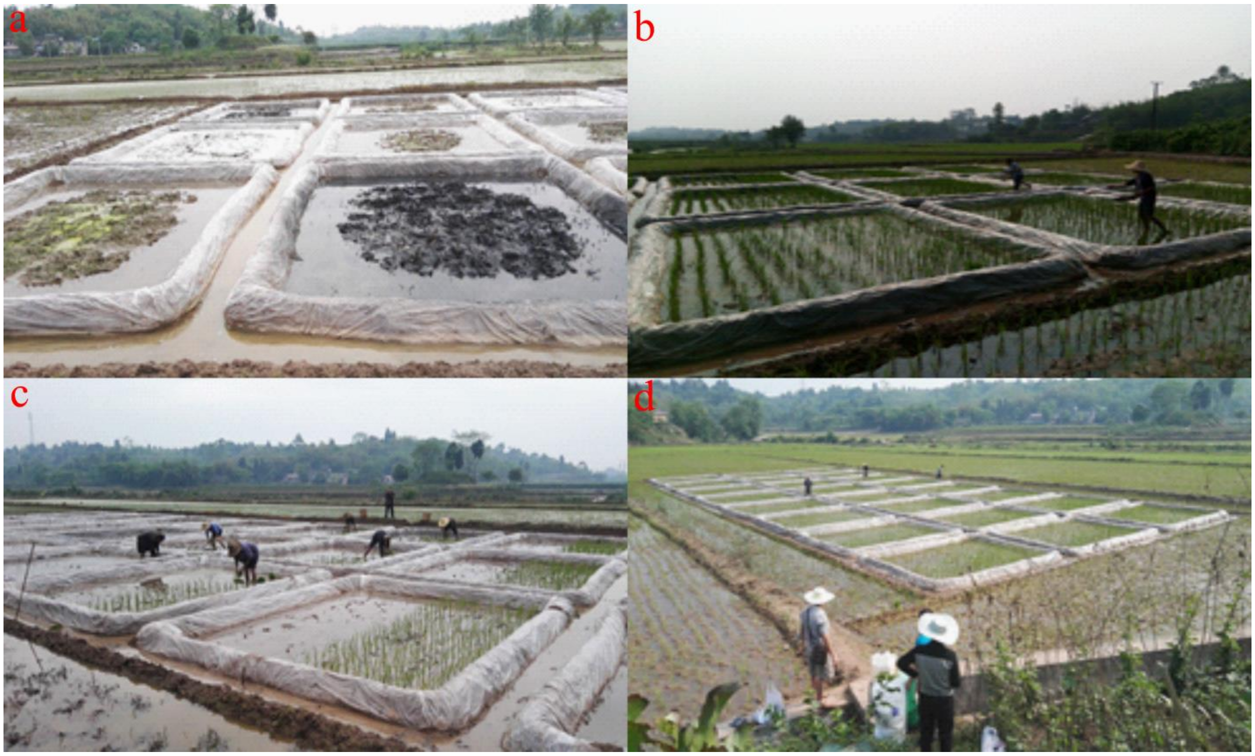

Figure S2. Sampling of Cd-contaminated soil from different layers of soil

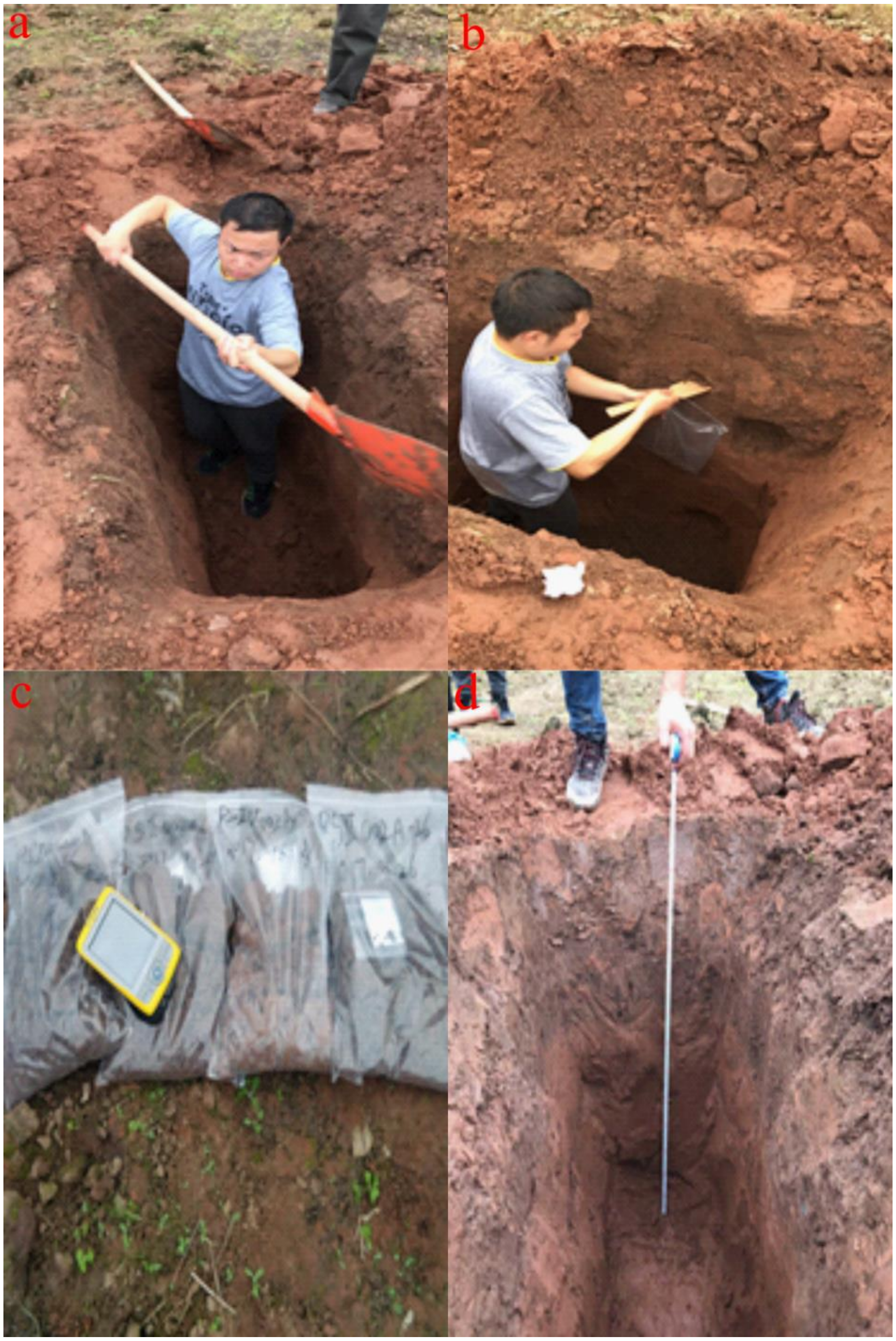

Figure. S3. Irrigation water sampling from different sources.

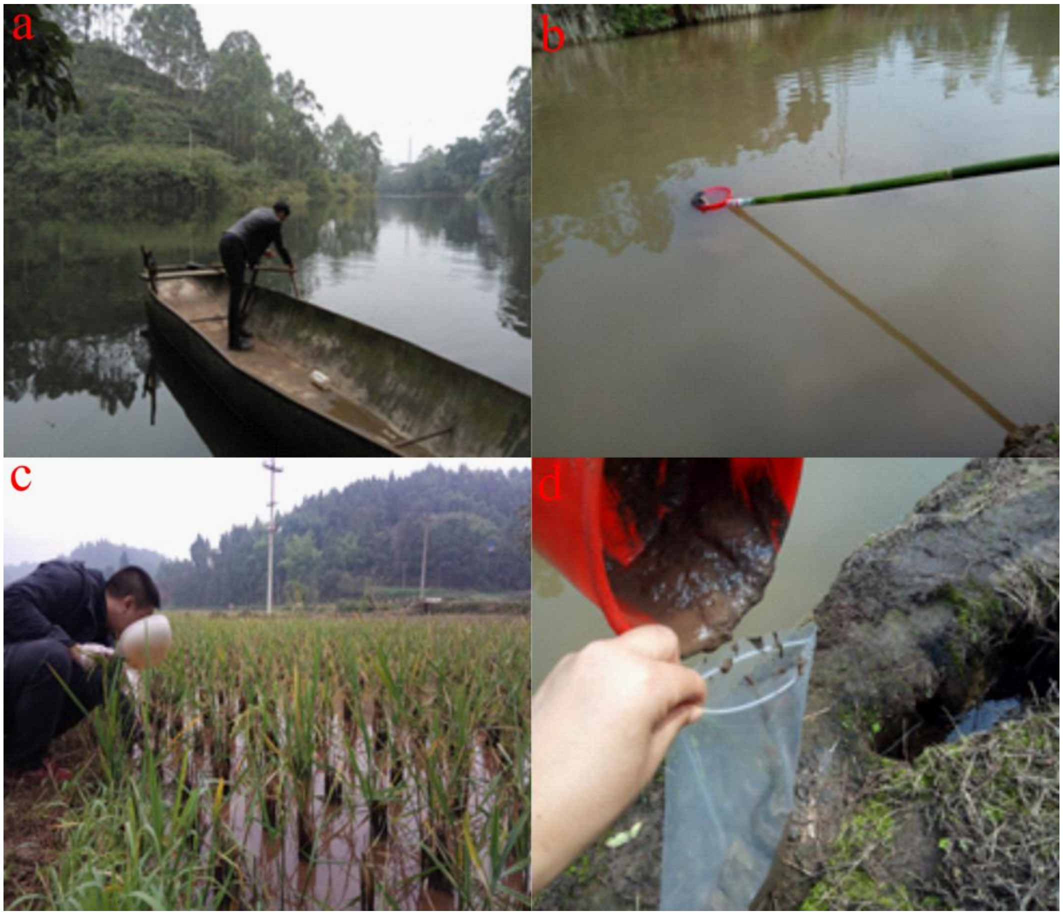

Figure S4. The sampling of wall mud, from a 100-year-old building.

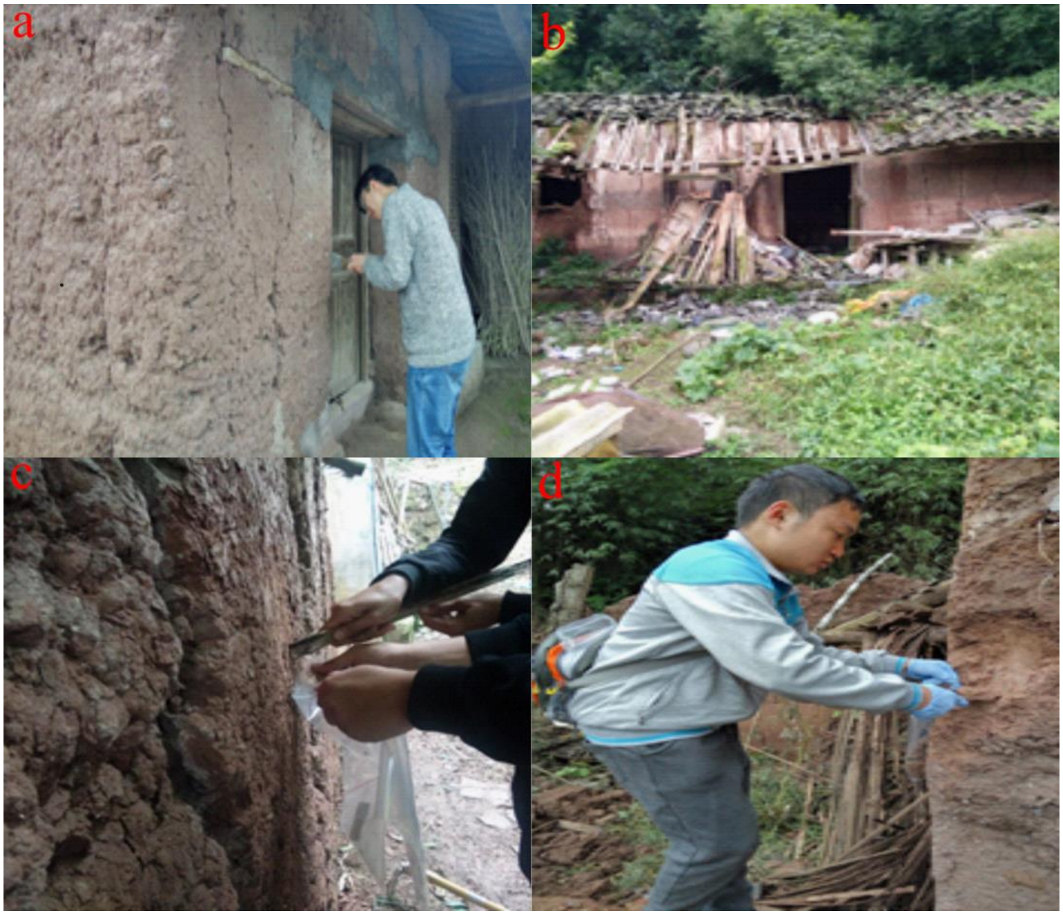

Figure S5. The soil depth and Cd concentration

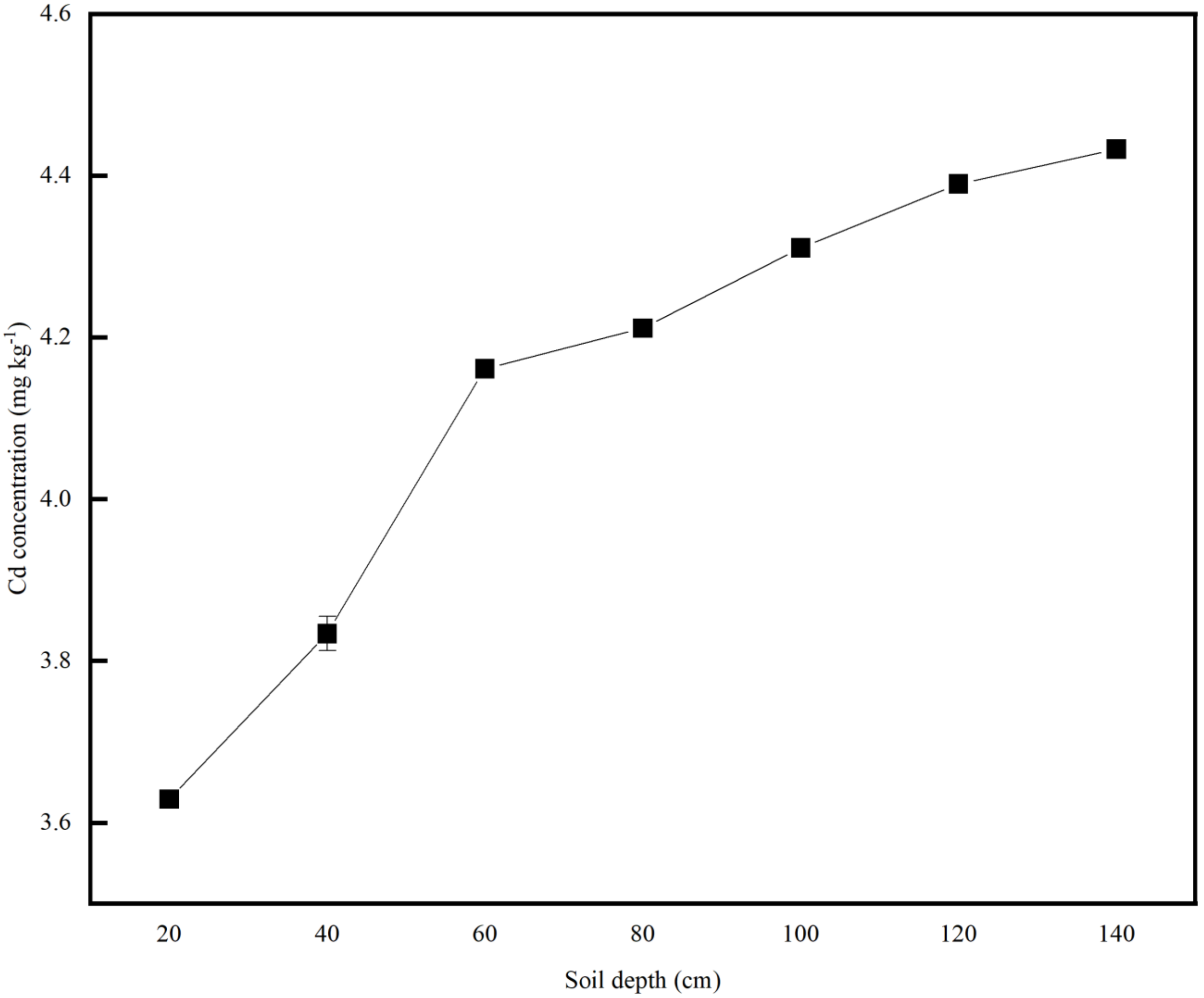

Supplement: Supplementary file 1 [file foods-12-00314-s001.zip › foods-2035857-supplementary.pdf]
